# Wood-Waste-Based Artificial Aggregates for Extrusion 3D-Printed Cementitious Composites: Hydration, Printability, and Mechanical Performance

**DOI:** 10.3390/ma19102013

**Published:** 2026-05-12

**Authors:** Fausta Kavaliauskienė, Vitoldas Vaitkevičius, Karolina Butkutė, Maris Sinka, Aleksandrs Korjakins

**Affiliations:** 1 Faculty of Civil Engineering and Architecture, Kaunas University of Technology, Studentų g. 48, 51367 Kaunas, Lithuania; vitoldas.vaitkevicius@ktu.lt (V.V.); karolina.butkute@gmail.com (K.B.); 2 Institute of Sustainable Building Materials and Engineering Systems, Riga Technical University, Kipsala Street 6A, LV1048 Riga, Latvia; maris.sinka@rtu.lv (M.S.); aleksandrs.korjakins@rtu.lv (A.K.)

**Keywords:** wood, lignocellulosic wastes, artificial aggregates, cement, 3D printing, recycling

## Abstract

This study investigates the feasibility of incorporating wood-based waste in cementitious composites for extrusion-based three-dimensional (3D) printing through the production of artificial aggregates. Because lignocellulosic residues can retard cement hydration, wood dust was chemically modified with a calcium nitrate-based accelerator and granulated into aggregates using disc granulation. The resulting aggregates were characterized for mechanical robustness, and their influence on cement hydration and microstructural development was evaluated using X-ray diffraction (XRD) and thermogravimetric/differential scanning calorimetry (TG/DSC). The modified aggregates were then incorporated into 3D printable cementitious mixtures to assess fresh-state properties, printability, and mechanical performance. The accelerator affected hydration by increasing bound water content and altering the development of hydration products. The produced aggregates exhibited sufficient crushing resistance for practical handling. The incorporation of artificial aggregates resulted in reduced compressive and flexural strengths compared to the reference mixture. However, the differences between mechanical properties measured in different loading directions were reduced, indicating a more uniform structural response in printed elements. The findings demonstrate that chemically treated wood-based aggregates can be successfully integrated into 3D printable cementitious systems, offering a promising pathway toward more sustainable construction materials.

## 1. Introduction

The construction sector is under increasing pressure to reduce its environmental impact, particularly because cement production has a high carbon footprint [1,2]. This has increased interest in more sustainable cementitious materials, including mixes that incorporate waste-derived components.

Lignocellulosic residues such as fine wood dust from furniture manufacturing are an abundant and largely underutilized waste stream [3,4]. The use of such fine fractions is particularly relevant, as their high specific surface area intensifies their interaction with the cementitious matrix and hydration processes. Incorporating wood dust into cement-based materials could reduce the demand for natural aggregates and support the valorization of industrial by-products [5,6]. However, wood-based additions are not straightforward to use in cementitious mixtures because they are often incompatible with cement hydration.

Wood particles contain water-soluble extractives (e.g., sugars) that can markedly retard hydration [7,8]. This effect is especially pronounced in very fine dust fractions, where the increased surface area enhances the release of these compounds into the pore solution, leading to delayed setting, reduced early strength, and weaker interfacial bonding. To mitigate these effects, the wood dust was chemically modified with a calcium nitrate solution to neutralize hydration-retarding compounds and improve its interaction with the cementitious matrix. Among available approaches, accelerating admixtures, such as calcium nitrate, have been reported to counteract hydration retardation and promote early-age reactions [8,9] while also enhancing the bond between organic particles and the surrounding matrix [10].

At the same time, extrusion-based 3D printing is gaining attention in construction because it can produce complex geometries without formwork and with less material waste [11,12]. For a cementitious mixture to be suitable for extrusion-based printing, it must exhibit a balance between flowability and structural build-up. Desirable fresh-state properties include continuous extrusion through the nozzle, stable filament formation, sufficient shape retention after deposition, and adequate buildability to support subsequent layers without collapse [13,14,15]. In contrast, insufficient flowability may lead to nozzle blockage or discontinuous extrusion, while excessive flowability may cause lateral spreading, loss of geometry, interlayer deformation, or structural instability. In the hardened state, desirable properties include sufficient compressive and flexural strength, good interlayer bonding, limited anisotropy, and controlled shrinkage deformation [16,17]. Lightweight or organic aggregates can strongly affect these requirements by changing rheology, water distribution, interfacial behavior, and dimensional stability [15,18].

Granulation offers a controlled route for producing artificial aggregates from waste materials. The resulting aggregates can be tailored in terms of size and internal structure, which makes them attractive for extrusion-based mixtures [19]. Nevertheless, when organic components such as wood dust are included, aggregate cohesion and bonding to the surrounding matrix become decisive for performance.

Although wood–cement composites and artificial lightweight aggregates have been studied extensively, little is known about combining chemically modified wood dust with artificial aggregates specifically for extrusion-based 3D printing. In particular, the links between the modification approach, aggregate microstructure, and printability-related performance remain poorly understood.

This study explored the use of very fine wood dust treated with calcium nitrate to produce artificial aggregates for cement-based mixtures. Although wood–cement composites and artificial lightweight aggregates have been widely studied, previous research has predominantly focused on wood fibers or relatively coarse particles [4,7,8]. In contrast, the use of very fine wood dust fractions remains comparatively less explored due to their high specific surface area and strong influence on cement hydration [18]. Moreover, in extrusion-based 3D printing, research has mainly focused on mineral or recycled aggregates, with limited attention given to lignocellulosic fine fractions [13,14]. The results show that these aggregates can provide satisfactory mechanical strength and physical properties, suggesting that they could be a viable alternative ingredient in certain cementitious formulations.

The research also examined how well the aggregates perform in extrusion-based 3D printing, focusing on hydration, microstructure development, and printability. Unlike conventional approaches that incorporate wood particles directly into cementitious mixtures, this study utilizes chemically modified fine wood dust integrated into artificial aggregates, enabling improved compatibility with the cementitious matrix and adaptation to extrusion-based processes. It examined how the modified wood-based aggregates influence cement hydration and the formation of the material’s internal structure and how these factors affect extrusion and shape stability during printing. Overall, the findings point to a promising approach to making construction materials more sustainable by reusing wood waste and improving its compatibility with cementitious binders, which may help reduce environmental impact.

## 2. Materials and Methods

### 2.1. Binders

In this study, two types of specimens were investigated: manually fabricated artificial aggregates (AAs) and 3D-printed samples (3DPSs). The binder used to produce AAs was CEM II/B-M (P-LL) 42.5 N cement supplied by the local manufacturer “Akmenės cementas” (Naujoji Akmenė, Lithuania). According to the manufacturer’s specifications, the cement consists of 65–79% Portland cement clinker, 21–35% main constituents, and 0–5% minor additional constituents. The composite composition of cement makes it suitable for sustainable cement-based systems, as partial clinker replacement reduces the overall clinker factor while maintaining mechanical performance. In addition to cement, burnt oil shale ash (BOSA) obtained from “Enefit Power AS” (Auvere, Estonia) was incorporated into the aggregate as a mineral component, contributing to the valorization of industrial by-products. The chemical compositions of CEMII/B-M (P-LL) 42.5 N and BOSA are shown in Table 1.

The specimens and 3D-printed elements with incorporated artificial aggregates were produced using a cement-based mixture designed for extrusion-based additive manufacturing. The mixture consists of a cementitious binder combined with mineral additives to enhance both fresh-state and hardened properties. The primary binder was CEM II/B-M (S-LL) 52.5 N cement, while the mixture also included sand (0–2 mm), fly ash, metakaolin, and chemical admixtures.

Although the exact formulation of the mixture is not fully disclosed due to proprietary reasons, the approximate composition, as provided by the manufacturer, is presented in Table 2. All component contents are given on a mass basis, as provided by the manufacturer. The detailed composition of the admixtures was also not disclosed.

### 2.2. Aggregate and Admixture

Artificial aggregates were produced using wood dust (WD) obtained from a local furniture factory. The dust was generated during the cutting and sanding of various wood-based boards (e.g., MDF, plywood, HPL, and HDF) and is typically collected as a mixed by-product that cannot be reused in production. Due to the presence of binders and resins originating from the processed boards, this material is generally classified as waste and must be transferred to licensed waste treatment facilities rather than being burned or disposed of on-site.

**Figure 1:**
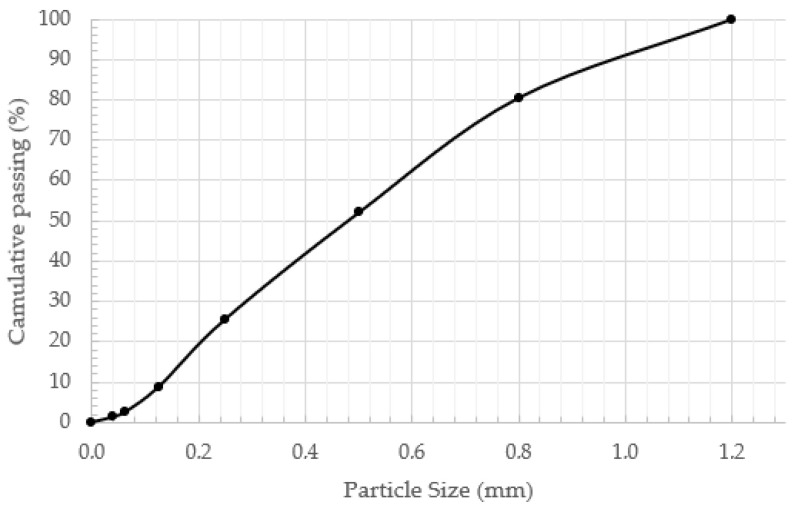
Wood dust particle size distribution.

The particle size distribution of the wood dust used in this study is presented in Table 3 and Figure 1. The material is mainly composed of particles in the 0.25–0.5 mm size range, which represents the dominant fraction. A noticeable proportion of coarser particles (1 mm) is also present, accounting for 19.5% of the total mass. The cumulative distribution curve shows a smooth trend with no visible gaps between size fractions, indicating continuous grading. The amount of particles smaller than 0.125 mm is limited, and the content of very fine particles (<0.063 mm) remains low. The gradual shape of the curve suggests that the material is distributed across multiple size fractions without abrupt changes, indicating a well-graded particle-size distribution in the wood dust used in the mixtures.

A calcium nitrate (Ca(NO_3_)_2_) accelerating admixture was incorporated during the production of AAs. The admixture was used to promote faster hydration of the cementitious binder and to shorten the initial setting time during granulation. The use of calcium nitrate also supports the production process by enabling more efficient granule formation and improving the overall integrity of the AAs at early stages.

### 2.3. Test Methods

#### 2.3.1. Granulation

**Figure 2:**
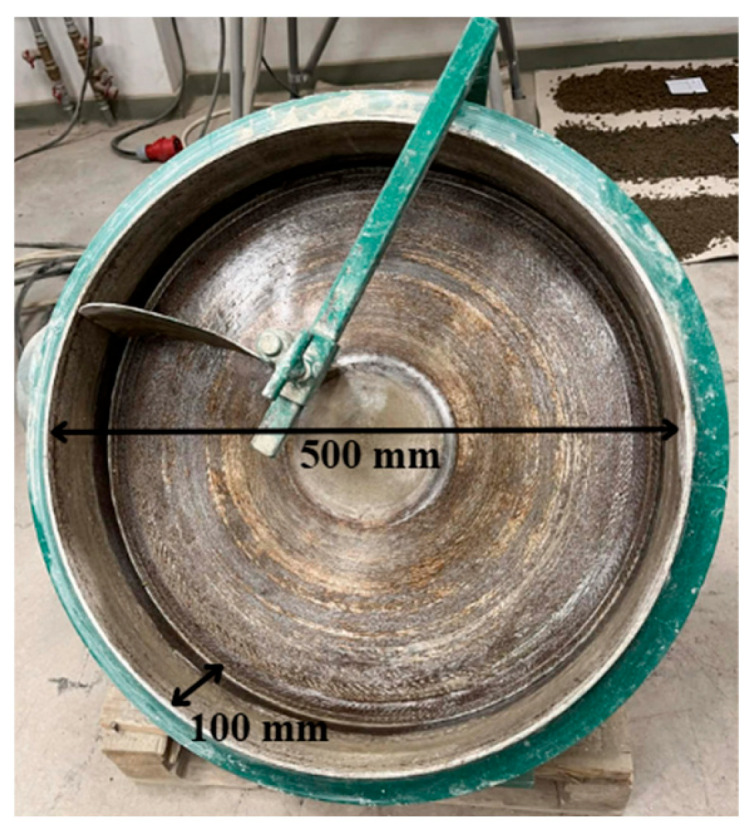
Laboratory-scale granulation disc.

In this research, artificial aggregates were made using a mechanical process, namely the granulation method [20]. The granulation process was performed using a laboratory-scale disc granulator (Figure 2). The disc had a diameter of 500 mm, a depth of 100 mm, an inclination angle of 45°, and a rotational speed of 35 rpm.

The production of artificial aggregates began by mixing dry materials together into the already rotating disc to ensure initial homogenization. After the dry materials were mixed, the liquid components (water premixed with accelerator) were gradually sprayed into the disc in a controlled manner. The total granulation process lasted approximately 5 min.

Following the granulation process, the produced artificial aggregates were left to dry under natural room conditions at 20 ± 2 °C and 50 ± 5% relative humidity for 48 h. After drying, the aggregates were sieved. In this study, only aggregates with a particle size range of 0–4.25 mm were used. Aggregates larger than 4.25 mm were excluded from further testing (around 30% of the whole volume). The selected aggregate size was determined based on the nozzle dimensions and operational requirements of the 3D printing system.

#### 2.3.2. Artificial Aggregate Crushing Strength

**Figure 3:**
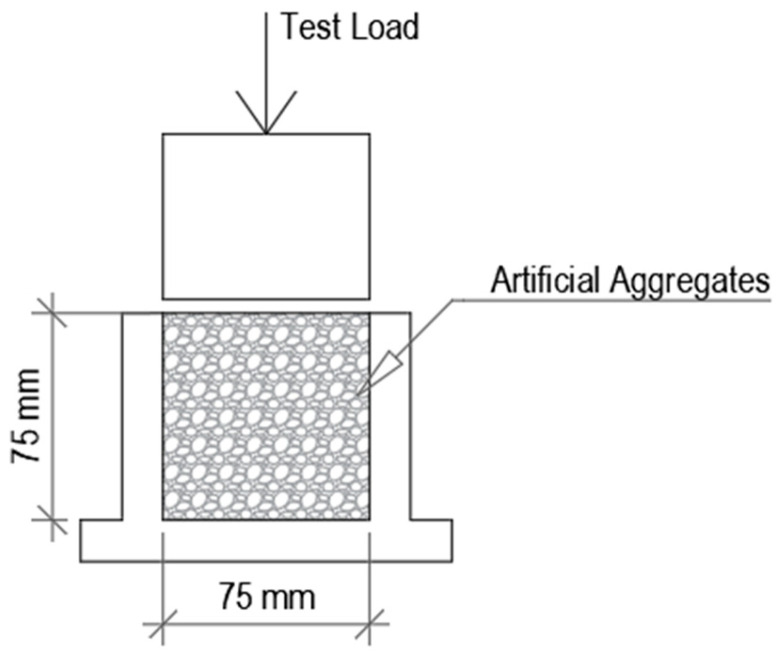
Artificial aggregate crushing scheme.

The mechanical performance of the aggregates was evaluated by determining their crushing resistance. The test was performed according to the EN 13055-1 standard [21]. A representative sample of aggregates was placed in a rigid steel cylinder, and a steadily increasing compressive load was applied using a testing machine until failure occurred (Figure 3). During loading, the force required to initiate and propagate particle breakage was recorded, and the maximum load sustained by the sample was used to assess aggregate strength. The crushing resistance of artificial aggregates was determined using 6 specimens.

#### 2.3.3. XRD Test

X-ray diffraction (XRD) analysis was conducted at the initial stage of the study to evaluate the interaction between cement CEM II/B-M (P-LL) 42.5 N and wood dust, both with and without the use of an accelerating admixture. The mineral composition of the materials was analyzed using a D8 Advance X-ray diffractometer (Bruker AXS GmbH, Karlsruhe, Germany). Prior to testing, the samples were left to dry under natural room conditions (at 20 ± 2 °C and 50 ± 5% relative humidity) and ground to obtain a homogeneous powder. The specimens were scanned over a 2θ range of 3–70° with a step size of 0.02°.

#### 2.3.4. TGA/DSC Test

Thermal analysis was conducted at the initial stage of the study to evaluate the interaction between cement and wood dust, both with and without the use of an accelerating admixture, as well as to assess changes in thermal behavior and mass-loss characteristics. Thermal analysis was performed using a Netzsch STA 409 PC Luxx analyzer (NETZSCH-Gerätebau GmbH, Selb, Germany)with ceramic sample handlers. Prior to testing, the samples were dried and ground to obtain a uniform particle size. The prepared material was then placed in crucibles and heated under controlled conditions to record mass changes and heat flow as a function of temperature. The samples were heated at a constant rate of 10 °C/min up to 1000 ± 3 °C in a nitrogen atmosphere under ambient pressure. The thermogravimetric (TG), derivative thermogravimetric (DTG), and differential scanning calorimetry (DSC) signals were recorded during the test, and the mass loss of the specimens was evaluated using the tangential method. XRD and TG/DSC analyses were performed as representative material characterization tests; therefore, no standard deviation was calculated for these measurements.

#### 2.3.5. SEM Test

The surface morphology and microstructural characteristics of the materials were examined using a high-resolution scanning electron microscope (SEM), Hitachi S-3400N (Hitachi High-Technologies Corporation, Tokyo, Japan). Prior to analysis, the samples were prepared and conditioned to ensure suitable surface quality for imaging.

The investigations were carried out on materials and specimens after more than 28 days of curing.

#### 2.3.6. Tests for Fresh State Composition

The workability of the 3D printing fresh mixtures was evaluated using the flow table test in accordance with EN 1015-3 [22]. This method is used to determine the consistency of cementitious mixtures by measuring their spread under dynamic loading.

In addition to the flow table test, the buildability of the mixtures was evaluated to assess their suitability for extrusion-based 3D printing. The test was performed by printing successive layers under constant process parameters until structural instability or collapse occurred. The maximum number of layers that could be deposited without significant deformation was recorded as an indicator of buildability. For each mixture, the flow table test was performed 6 times.

#### 2.3.7. Three-Dimensional Printer

Concrete specimens were produced using a three-dimensional printer developed at Riga Technical University (RTU) for the fabrication of cement-based elements. The system allows printing of objects with dimensions up to approximately 1500 × 1000 × 1000 mm and is equipped with a material container with a capacity of about 30 L.

**Figure 4:**
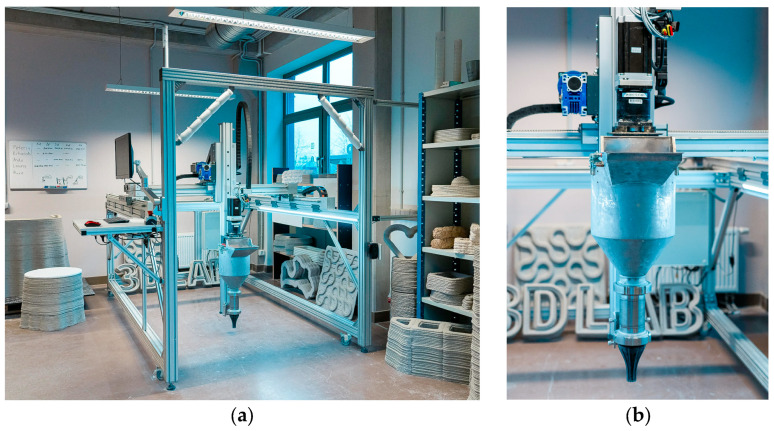
Three-dimensional printer (**a**) and the nozzle (**b**).

The material was extruded through a nozzle (Figure 4b) with a diameter of 25 mm, resulting in layers with a width of approximately 35–45 mm and an average height of around 10 mm. The rheological properties of the mixture played a key role in ensuring proper extrusion, shape retention, and buildability of the printed layers. During printing, the stability of the deposited layers was visually monitored to ensure consistent geometry and adequate interlayer bonding. The printer setup is shown in Figure 4a.

#### 2.3.8. Three-Dimensional Printing and Curing

All mixing, printing, and curing steps were carried out under controlled laboratory conditions. During the entire process, the temperature was maintained at 20 ± 2 °C, and the relative humidity was approximately 30 ± 5%.

The mixtures were prepared using a portable mixer, Rubimix-9 (Rubi UK Ltd., Rainham, UK), operating at approximately 780 rpm.

First, all dry components were weighed and combined. The dry materials were then briefly mixed (10–15 s) to ensure an even distribution. After this initial step, tap water at a temperature of 9–10 °C was added, and the mixture was mixed for a further 100–120 s until a uniform consistency was achieved.

Part of the mixture was used for testing fresh properties and for casting specimens, while the remaining material was transferred into the printer hopper. The mixture was extruded through the nozzle until a stable and continuous flow was obtained. Elements measuring 160 × 160 × 200 (h) mm and 160 × 160 × 120 (h) mm were printed at a speed of 100 mm/s.

**Figure 5:**
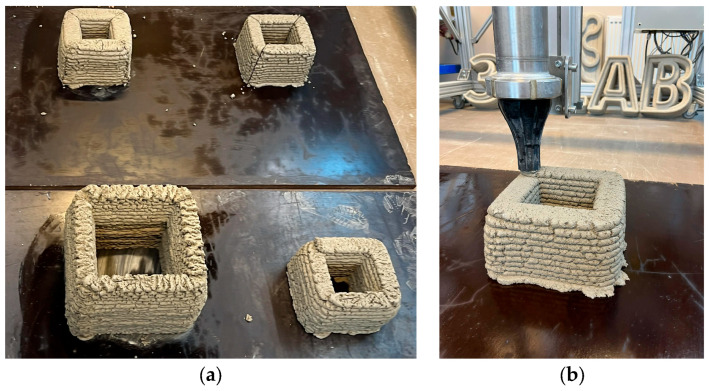
The 3D-printed specimens (**a**) and the extrusion process with the printing nozzle (**b**).

After printing, all specimens (Figure 5a) were kept under the same environmental conditions for 28 days. Once cured, the printed elements were cut into smaller samples using a concrete angle grinder for mechanical testing.

#### 2.3.9. Tests for Printed Objects

After adding water, the prepared mixtures were divided into two portions. One portion was used to cast specimens in molds in accordance with the EN 1015-11 standard [23], while the remaining material was transferred to the printer hopper and used for the fabrication of 3D-printed elements.

**Figure 6:**
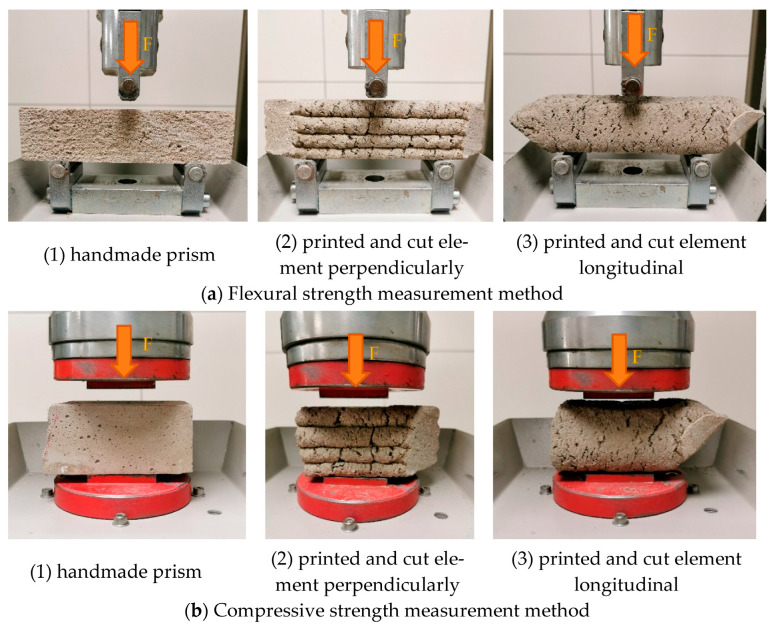
Flexural and compressive strength measuring methods [24].

All specimens were cured under identical laboratory conditions for 28 days. After the curing period, the printed elements were cut into smaller samples using a concrete angle grinder. Both the molded prisms and the extracted samples from the printed elements were then subjected to mechanical testing. The testing directions used for the flexural and compressive strength measurements of the molded and printed specimens are illustrated in Figure 6 [24]. The flexural and compressive strengths were determined using a compression and bending testing machine, Ratio TEC. Flexural strength was determined using 3 specimens, while compressive strength was determined using 6 specimens for each mixture and testing direction.

#### 2.3.10. Shrinkage Measurement

**Figure 7:**
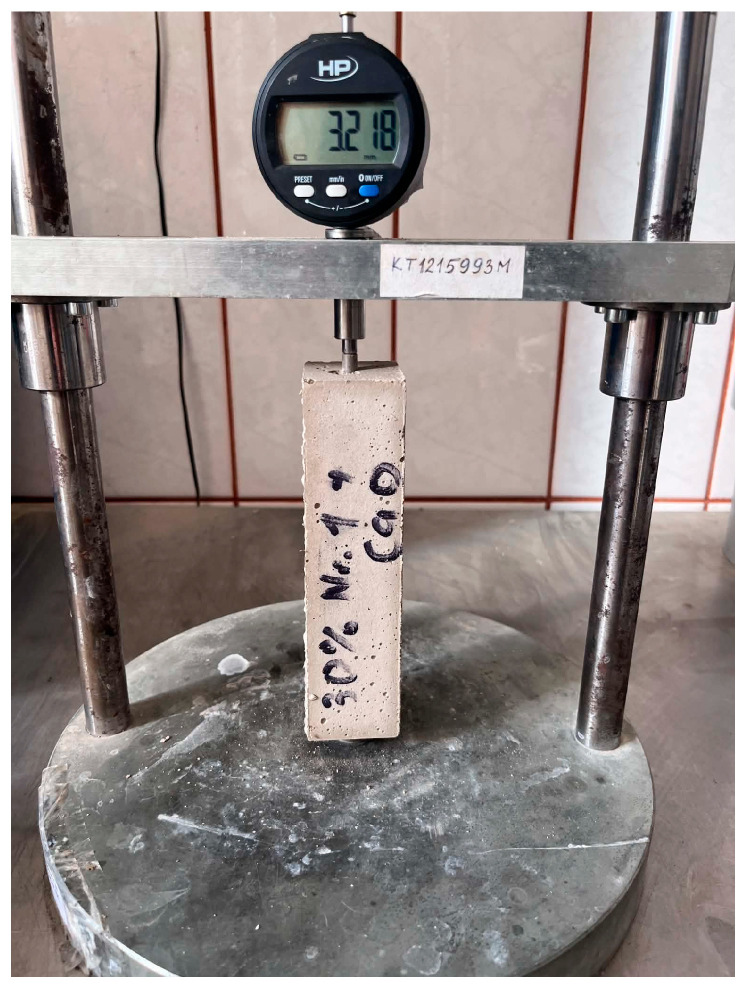
The shrinkage measurement setup.

Shrinkage measurements were performed using 40 × 40 × 160 mm prismatic specimens. After 24 h of curing, the specimens were demolded, and the first length measurement was recorded as the initial reference value. The length changes were then monitored periodically during the curing period from 1 to 290 days. For each mixture, shrinkage values were calculated by averaging the results from six specimens. The shrinkage deformation was calculated as the relative change in specimen length from the initial length. The shrinkage measurement setup is shown in Figure 7.

The shrinkage deformation was expressed as follows:

(1) $$D_{t}=\frac{L_{0}-L_{t}}{L_{0}}\times 100$$

where $D_{t}$ is the shrinkage deformation (%), $L_{0}$ is the initial specimen length measured after 24 h of curing, and $L_{t}$ is the specimen length measured at the selected curing age t.

## 3. Results

### 3.1. XRD Test Results

To assess the effect of the accelerator on the phase composition of the cementitious system, X-ray diffraction (XRD) analysis was performed on test mixtures prepared both with and without the accelerating admixture. The compositions of these mixtures are presented in Table 4. Both mixtures were designed with the same base components, differing only in the inclusion of a calcium nitrate-based accelerator in one of them, enabling a direct and consistent comparison.

The test mixture consisted of Portland cement, wood dust, and water, while the second mixture additionally included the accelerating admixture. This approach enabled isolating the influence of the accelerator and evaluating its impact on the formation of hydration products and the development of crystalline phases.

**Figure 8:**
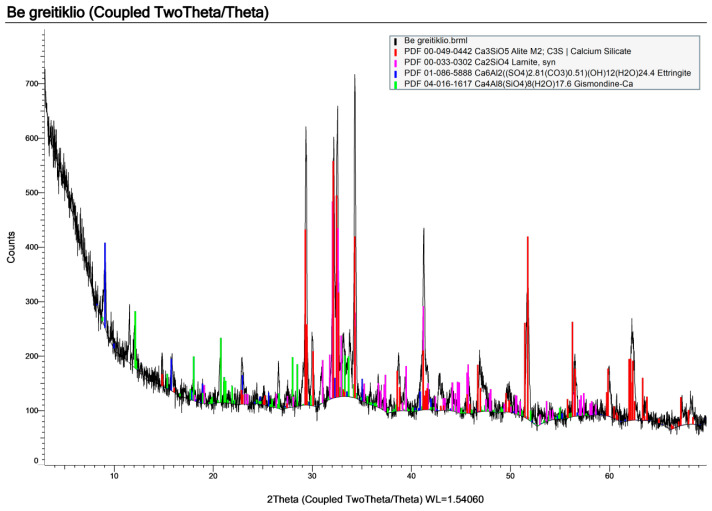
XRD pattern of the sample without an accelerator.

The test mixture without the accelerator (Figure 8) shows the expected cement-related phases, including residual alite (C_3_S), belite/larnite (C_2_S), and ettringite. The diffractogram, however, shows relatively low peak intensities on an elevated background. A broad diffuse hump in the ~28–36° 2θ range is also visible, commonly attributed to a substantial fraction of poorly crystalline and/or X-ray–amorphous hydration products. Together, these features indicate a lower overall crystallinity and suggest that hydration is less advanced at the time of testing.

**Figure 9:**
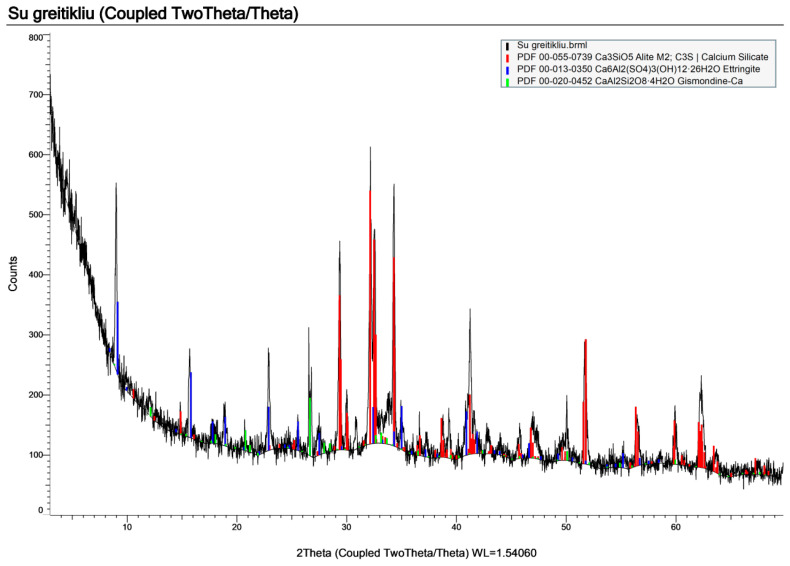
XRD pattern of the sample with accelerator.

In contrast, the mixture containing the accelerator (Figure 9) shows noticeably stronger and sharper reflections, particularly in the ~29–35° 2θ region associated with calcium silicate phases. Low-angle ettringite peaks are also more pronounced. The higher peak-to-background ratio, therefore, points to a higher degree of crystallinity and a more advanced formation of crystalline hydration products within the same curing period.

While the qualitative phase assemblage appears broadly similar in both mixtures, the accelerator clearly enhances hydration progress and results in a more developed binder structure over the same time frame. The non-accelerated mixture appears less mature, consistent with slower hydration kinetics and potentially reduced early-age performance unless longer curing is provided.

### 3.2. TG/DSC Test Results

**Figure 10:**
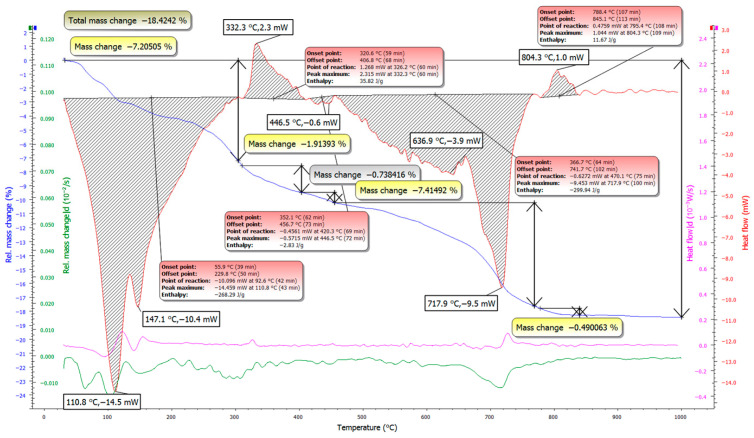
TG/DSC curves of the sample without accelerator.

**Figure 11:**
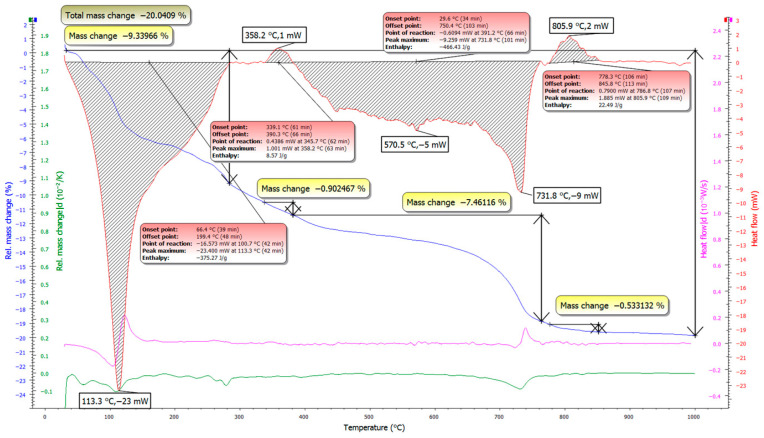
TG/DSC curves of the sample with accelerator.

Thermal analysis (TG/DSC) of the samples with and without the accelerator is shown in Figure 10 and Figure 11, and the corresponding mixture compositions are listed in Table 4. The two systems exhibit similar thermal behavior, with mass loss occurring over similar temperature intervals. To provide a clearer basis for comparison, the main mass-loss regions were attributed to the dehydration of physically and chemically bound water, portlandite-related decomposition, and carbonate decomposition.

The test mixture containing the accelerator exhibits a higher total mass loss (20.04%) compared to the mixture without the accelerator (18.42%), indicating a greater amount of thermally decomposable hydration-related products under the same testing conditions. However, this result should be interpreted as an indicative comparison rather than as a direct quantitative determination of individual hydration phases.

The accelerator-containing mixture shows a higher total mass loss (20.04%) than the mixture without accelerator (18.42%). Under identical testing conditions, this is consistent with a higher amount of thermally decomposable hydration-related constituents, mainly bound water associated with hydration products, and therefore with a more advanced hydration state in the accelerated system.

In the low-temperature region (~100–200 °C), both mixtures exhibit endothermic effects related to the release of physically and weakly bound water and the dehydration of early hydrates. This response is more pronounced for the accelerator-containing mixture, indicating a higher bound-water contribution and supporting more intensive early hydrate formation.

A pronounced difference is observed in the intermediate temperature range (~300–400 °C). This region is often associated with portlandite-related dehydration, although contributions from other hydrates may overlap depending on the binder chemistry and test conditions. The mixture with accelerator shows a stronger, well-defined feature in this range with higher associated mass loss, suggesting differences in hydration product development between the two systems. Since full quantification of portlandite was not performed, this observation is presented as a comparative trend rather than a precise phase-content measurement. This points to a reduced relative contribution of portlandite when the accelerator is used, which may result from suppressed CH formation and more efficient consumption of CH in secondary reactions—both consistent with the development of a denser and more mechanically efficient hydrate assemblage.

At higher temperatures (~600–750 °C), both mixtures show mass loss related to carbonate decomposition. Differences here are minor; however, the accelerator-containing system displays a slightly more uniform response, suggesting broadly similar decomposition chemistry.

Overall, the TG/DSC results support that the accelerator promotes reaction development. The stronger bound-water-related signal at low temperatures and the observed differences in the portlandite-related temperature region are both consistent with a more advanced hydration-related response.

When these observations are combined with the XRD results, the same trend is observed. The accelerated mixture shows sharper, more intense phase reflections, along with a higher bound-water contribution, while TG/DSC results indicate differences in the thermally decomposable hydration-related phases. In contrast, the non-accelerated mixture appears less progressed at the same curing age, with weaker hydrate-related signals and less favorable phase development. Taken together, the results indicate that the accelerator is beneficial for accelerating hydration and improving microstructural development. However, the interpretation remains based on comparative XRD and TG/DSC evidence rather than full quantitative phase analysis.

### 3.3. Artificial Aggregate Test Results

**Figure 12:**
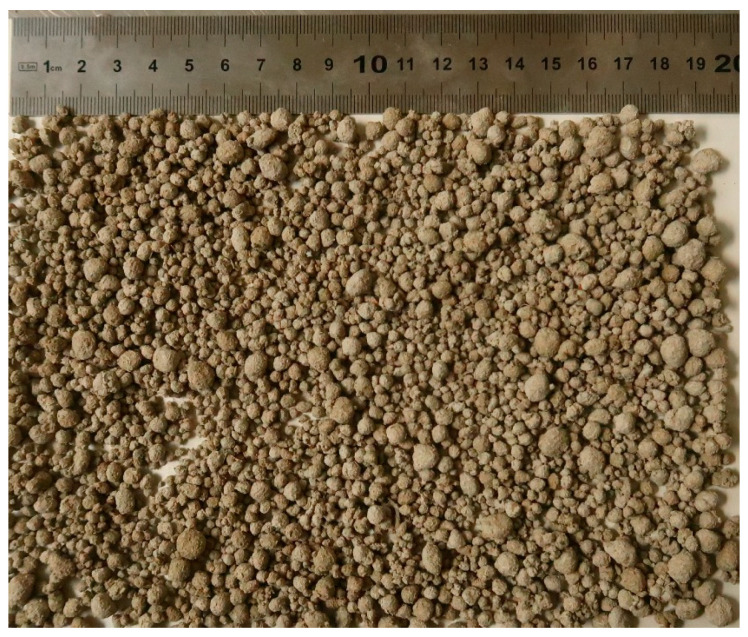
AA1 granules.

The results of the study are presented in this section, beginning with the development of artificial aggregates. After evaluating the obtained aggregate properties, suitable binder materials were chosen for granule production. The mixture compositions are given in Table 5, while the appearance of the produced granules is shown in Figure 12.

The selected composition was designed to ensure sufficient binding capacity while incorporating waste-based materials. Cement served as the primary binder, while wood dust and burnt oil shale ash were used as lightweight components to replace conventional raw materials and thereby reduce the CO_2_ footprint. The water content and accelerator dosage were adjusted to support stable granulation and promote early strength development. A single aggregate composition was investigated to assess the feasibility of the proposed approach and to examine its behavior in more detail.

The mechanical performance of the artificial aggregates was evaluated with a crushing strength test. **The granules failed at a maximum load of 8.2 kN**. The obtained crushing resistance can be considered promising for the intended application, as artificial aggregates of comparable waste-derived material origin and similar production approach have been reported in the literature [24] to exhibit crushing resistance values of approximately 4.95–10.05 kN. This indicates that the produced wood-based artificial aggregates fall within the performance range of similar artificial aggregates and possess sufficient mechanical robustness for handling, mixing, and incorporation into extrusion-based 3D printable cementitious composites.

The produced artificial aggregates were sieved before further use because the granulation process naturally produces particles with a range of sizes rather than a single, uniform fraction. Since the material was intended for extrusion-based 3D printing, the maximum aggregate size was set to **4.25 mm** to avoid nozzle blockage and to ensure smooth, stable extrusion. Any particles larger than this limit were therefore removed.

**Figure 13:**
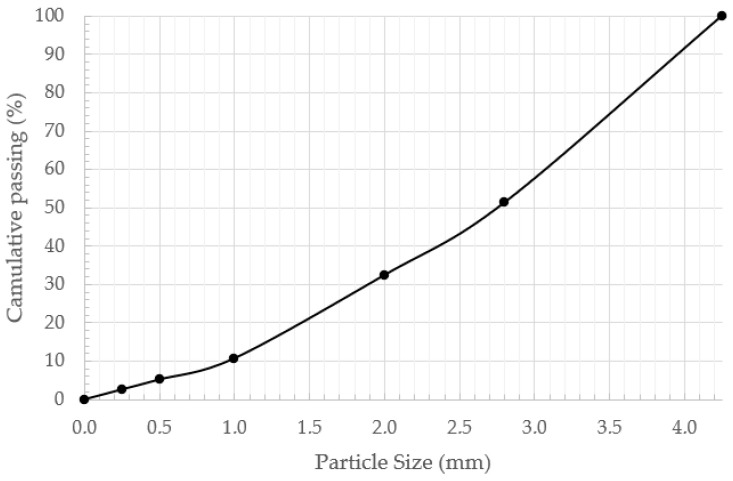
Artificial aggregate particle size distribution.

After sieving, about 30% of the produced aggregates were excluded from the mix design and were not used in the subsequent experiments. This also means that the reported test results reflect the behavior of the selected, print-compatible fraction rather than the full, as-produced batch. The particle-size distribution of the retained fraction is shown in Table 6 and Figure 13, which illustrates the grading of aggregates used throughout the study.

### 3.4. SEM Test Results

Representative SEM images of the developed composites are presented and discussed in this section. The micrographs illustrate the morphology and distribution of the constituent phases, including the cement matrix, wood particles, and burnt oil shale ash.

**Figure 14:**
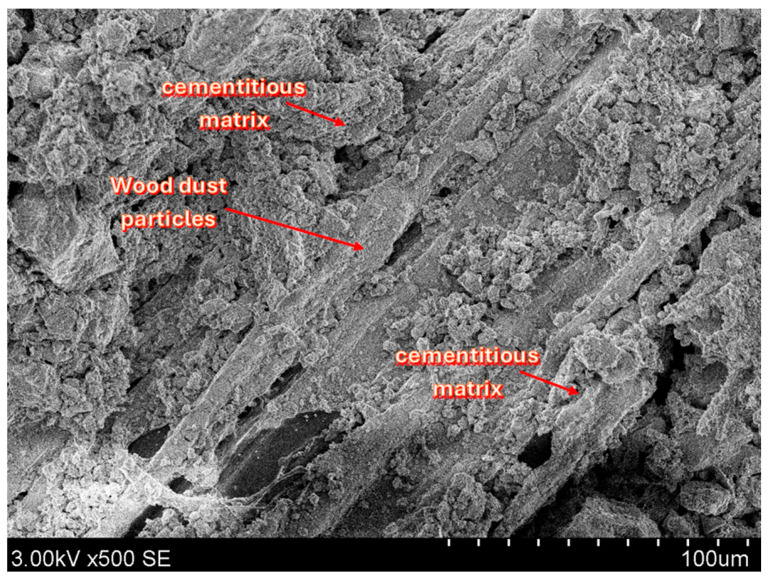
SEM image of AA1 (×500).

Figure 14 shows the microstructure of the produced AAs. The wood dust is easy to recognize from its fibrous, cellulose-like appearance, and many fibers are covered by a thin layer of cement hydration products mixed with burnt oil shale ash-derived material. The aggregate appears uniform, with the individual particles well bonded into a coherent structure.

**Figure 15:**
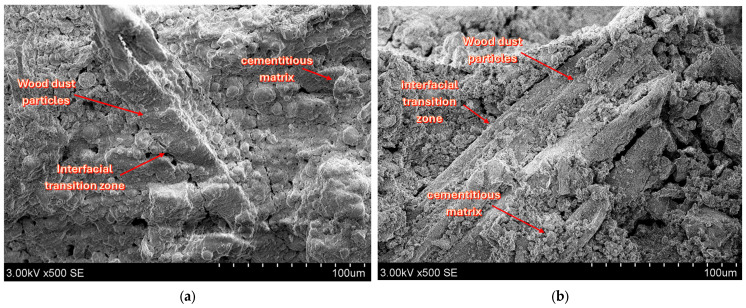
SEM images of cracked AA1: (**a**,**b**) show fractures in wood dust particles.

Most SEM images show a uniform internal structure in the produced aggregates, although some damaged particles (cracked or partly disintegrated) were also observed. In Figure 15, wood dust particles can be seen locally separating from the surrounding matrix. This separation may be associated with shrinkage within the aggregate, as evidenced by visible microstructural features.

Cracks were also often observed to run along the wood dust particles or cut through them, suggesting that these areas provide easier routes for fracture to develop. This is consistent with a weaker interfacial transition zone (ITZ) between the organic particles and the cementitious matrix, together with the higher porosity and greater deformability of the wood inclusions. Differences in physical and mechanical properties between the organic and mineral phases may also promote local stress concentrations and trigger microcrack formation during drying and hardening.

### 3.5. Three-Dimensional Printing

Three different mixture compositions were used for the 3D printing experiments, namely 3DREF, 3D30, and 3D30+2 (Table 7). The reference mixture (3DREF) was prepared without the incorporation of artificial aggregates, while the 3D30 mixture contained 30% artificial aggregates by volume. In the 3D30+2 mixture, the same amount of AAs was used, with the addition of quicklime (CaO) as a supplementary component. The addition of quicklime (CaO) was intended to reduce shrinkage by acting as an expansive component during the hydration process [25,26].

All mixtures were successfully processed using the three-dimensional printing system, enabling further analysis of their properties in the hardened state.

### 3.6. Three-Dimensional Printing Results

The fresh-state properties of the mixtures used for three-dimensional printing were evaluated in order to assess their suitability for the printing process. Particular attention was given to parameters influencing workability, flow behavior, and density of the mixtures. The results are presented in Table 8. It was observed that water content significantly affected printability. Therefore, the amount of added water was adjusted during the experiments to achieve stable extrusion under consistent printing conditions.

Although no rheometer-based measurements were conducted in this study, the printability of the mixtures was evaluated based on their extrusion behavior, shape retention, and the visual stability of the deposited layers. All mixtures were extruded smoothly through the 25 mm nozzle, with no interruptions or nozzle blockages. The printed filaments maintained their shape after deposition, and no significant interlayer deformation or collapse was observed during the laboratory-scale printing. Mixtures containing artificial aggregates required slightly more water, which is likely related to the water absorption capacity of the organic-based aggregate particles.

In previous studies [16,24] using the same RTU laboratory-scale 3D printing system, printable mixtures with artificial aggregates showed consistency/flow values of approximately 16–17 cm, while mixtures with waste- and organic-based artificial aggregates showed a wider flow range of 14.6–18.0 cm. Therefore, the flow table results obtained in the present study (15.2–15.7 cm) fall within a comparable range and confirm that the developed mixtures had suitable consistency for extrusion-based printing.

The mechanical properties of the investigated composites are summarized in Table 9, including bending and compressive strength results for 3D-printed and extracted specimens (longitudinal and perpendicular directions), as well as for cast prisms.

In general, cast prisms showed the highest strength. For the reference mixture (3DREF), the compressive strength was 82.8 MPa, and the bending strength was 6.04 MPa. The 3D-printed specimens yielded lower results, as expected for extrusion-based printing, because the material is built layer by layer, and the interfaces between layers can be weaker.

To more accurately evaluate the influence of different mixtures on mechanical properties, a statistical analysis of the data was performed by comparing the mean compressive and flexural strengths of the 3DREF, 3D30, and 3D30+2 mixtures. For this purpose, analysis of variance was applied. The normality assumption was checked using the Shapiro–Wilk test, while the homogeneity of variances for the strength characteristics among the mixtures was assessed using Levene’s test. Since these assumptions were not satisfied for all mixtures, the Kruskal–Wallis rank-sum test was also applied. The results of the statistical analysis are presented in Table 10.

The values presented in the table are the *p*-values of the statistical tests. The assumptions of normality and homogeneity of variances required for analysis of variance were satisfied in all cases, except for the compressive strength in the perpendicular direction for the 3DREF mixture. In this case, the obtained *p*-value of 0.0283 indicates that the normality assumption was not satisfied; therefore, the comparison of mixture strength should be based on the non-parametric Kruskal–Wallis rank-sum test. In the remaining cases, the one-way ANOVA test, which has greater statistical power, was considered appropriate.

Based on these tests, the compressive strength of the mixtures differed significantly in both the perpendicular and longitudinal directions, while the flexural strength differed significantly only in the perpendicular direction. However, the statistical test did not indicate a significant difference (*p*-value = 0.223) in flexural strength in the longitudinal direction. This result may be related to the limited sample size, and a larger number of specimens could potentially reveal statistically significant differences.

For the 3DREF mixture, a noticeable difference between longitudinal and perpendicular compressive strengths indicates some degree of anisotropy. However, for mixtures 3D30 and 3D30+2, the differences between longitudinal and perpendicular compressive strength are relatively small. This suggests that the bonding between printed layers was relatively strong, resulting in more uniform mechanical behavior in different directions. A similar trend is observed in the bending strength results. The cast prisms consistently yielded higher values than the printed specimens.

Using the modified aggregates (3D30 and 3D30+2) resulted in lower compressive and bending strengths than those of the reference mixture. This is likely because the AAs are less stiff and not as strong, and they also change how the material is structured internally. On the positive side, these mixes behaved more similarly in different directions, which is an advantage in 3D printing, where uniform performance is important regardless of loading orientation.

### 3.7. Shrinkage Measurement Results

**Figure 16:**
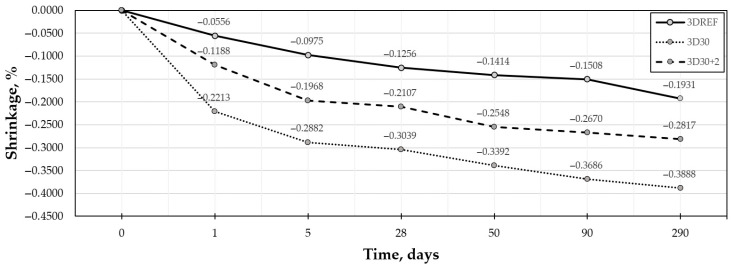
Shrinkage behavior of 3D-printed concrete specimens.

The shrinkage behavior of the developed mixtures was analyzed to evaluate their dimensional stability during curing. This aspect is especially relevant for mixtures containing artificial aggregates produced from organic-origin wood waste. Three different mixture compositions, presented in Table 7, were investigated, and the results are shown in Figure 16. In addition, the possible effect of CaO on reducing shrinkage deformation was evaluated.

All mixtures showed a steady increase in shrinkage over time, but the amount of shrinkage varied strongly with mixture composition. The reference mixture, 3DREF, had the lowest shrinkage throughout the entire test period. After 1 day, its shrinkage was −0.0556%, and it slowly increased to −0.1931% after 290 days.

The mixture with artificial aggregates (3D30) showed the highest shrinkage over the entire measurement period. Shrinkage increased quickly during the first days of curing, reaching −0.2213% after 1 day and −0.2882% after 5 days. After this early stage, the rate of increase became slower, and the shrinkage reached −0.3888% after 290 days. Compared with 3DREF, the final shrinkage of 3D30 was about twice as high. This shows that using artificial aggregates made from organic wood waste increased the tendency for dimensional changes during curing.

Adding CaO clearly reduced shrinkage. The 3D30+2 mixture had lower shrinkage than 3D30 at all ages. After 1 day, the shrinkage of 3D30+2 was −0.1188%, compared with −0.2213% for 3D30. The same pattern was seen at later ages, and after 290 days, the shrinkage of 3D30+2 reached −0.2817%, which is about 28% lower than that of 3D30. Even so, the shrinkage of 3D30+2 was still higher than that of the reference mixture.

In summary, the results show that the use of artificial aggregates increased shrinkage compared with the reference mixture. However, adding CaO reduced this effect and improved the dimensional stability of the mixture with artificial aggregates. This suggests that CaO can be a useful additive for limiting shrinkage in cement-based composites that contain organic-origin artificial aggregates.

## 4. Conclusions

This study investigated the feasibility of using artificial aggregates produced from fine wood waste in extrusion-based 3D printable cementitious composites. The main findings are summarized as follows:

- Artificial aggregates were successfully produced from fine wood waste using a granulation process. The resulting particles demonstrated adequate mechanical strength for handling, mixing, and use in cement-based mixtures. These results indicate that fine wood waste can be effectively repurposed as a functional component in 3D-printable composite materials.
- The calcium nitrate-based accelerator improved the hydration behavior of the wood–cement system. XRD and TG/DSC results indicated a more developed hydration response in the accelerated mixture, suggesting improved compatibility between the organic particles and the cementitious matrix.
- The incorporation of artificial aggregates reduced the density of the printable mixtures while maintaining suitable fresh-state properties for extrusion. This shows that wood-waste-based artificial aggregates can be used as a lightweight component in 3D printable cementitious composites.
- The mixtures containing artificial aggregates had lower compressive and flexural strength compared with the reference mixture. However, the differences between the longitudinal and perpendicular testing directions were reduced, indicating a more uniform mechanical response of the printed elements. This suggests that artificial aggregates may contribute to reduced anisotropy in 3D-printed composites.
- Shrinkage measurements showed that the mixture containing artificial aggregates had higher deformation than the reference mixture. The addition of CaO reduced this effect, indicating its potential to improve the dimensional stability of mixtures containing organic artificial aggregates.
- Overall, the results demonstrate that chemically modified wood waste artificial aggregates can be incorporated into extrusion-based 3D printable cementitious composites. This approach supports the valorization of fine wood waste and may contribute to the development of more sustainable lightweight construction materials. However, the study was limited to laboratory-scale testing and initial material characterization. Further research should focus on long-term durability, water absorption, biological resistance, full-scale printing performance, and the mechanisms responsible for the reduced anisotropy observed in printed elements.

## Figures and Tables

**Table 1:** Chemical compositions of Portland cement and burnt oil shale ash.

Components	Quantity, %	
	CEMII/B-M (P-LL)	BOSA
CaO	57.5	37.81
SiO_2_	18.4	29.5
SO_3_	3.42	10.38
Al_2_O_3_	3.29	6.52
Fe_2_O_3_	3.03	2.79
MgO	2.90	4.0
K_2_O	0.88	3.34
TiO_2_	0.24	0.35
Na_2_O	0.13	0.26
P_2_O_5_	-	0.18
Cl	-	0.48

**Table 2:** Composition of commercial 3D concrete mixture.

Component	Content by Mass (%)
Cement CEM II/B-M (S-LL) 52.5 N	20
Sand (0–2 mm)	66
Fly ash	5
Metakaolin	8
Chemical admixtures	1
Binder-to-sand ratio	0.30
Water-to-binder ratio	0.55
Water-to-cement ratio	0.90

**Table 3:** Particle size distribution.

Material	Sieve Mesh Diameter, mm and Residue in %							
	1.2	0.8	0.5	0.25	0.125	0.063	0.04	0.00
Wood dust	0	19.5	28.5	26.4	16.8	6.2	1.3	1.3

**Table 4:** Composition of test specimens with and without an accelerator.

Materials	Specimen’s Name and Amount of Materials (kg/m^3^)	
	Without Accelerator	With Accelerator
Portland Cement CEM II	435	345
Wood Dust	260	210
Water	305	240
Calcium Nitrate accelerator	-	205

**Table 5:** Artificial aggregate composition.

Materials	Artificial Aggregate Name and Amount of Materials (kg/m^3^)
	AA1
Portland Cement CEM II	165
Burnt Oil Shale Ash	335
Wood Dust	165
Water	210
Calcium Nitrate accelerator	125

**Table 6:** Artificial aggregate particle size distribution.

AA Name	Sieve Mesh Diameter, mm and Residue in %						
	4.25	2.8	2.0	1.0	0.5	0.25	0.00
AA1	0.0	48.7	18.9	21.7	5.5	2.6	2.6

**Table 7:** Three-dimensional printing composite composition.

Materials	Composite Name and Materials Amount (%)		
	3DREF	3D30	3D30+2
Commercial 3D concrete mixture	100	70	68
Artificial Aggregate AA1	-	30	30
Quicklime (CaO)	-	-	2

**Table 8:** Fresh 3D printing composite parameters.

Composite Number	Water Amount L/kg	Flow Table Results, cm	Bulk Density, kg/m^3^
3DREF	0.09	15.2	2125
3D30	0.11	15.2	1870
3D30+2	0.12	15.7	1890

**Table 9:** The analysis results of 3D-printed and cast prisms.

Composite Number	3D-Printed and Cut Elements				Cast Prisms	
	Longitudinal		Perpendicular		Bending Strength (MPa)	Compressive Strength (MPa)
	Bending Strength (MPa)	Compressive Strength (MPa)	Bending Strength (MPa)	Compressive Strength (MPa)		
3DREF	4.81	45.9	5.29	57.0	6.04	82.8
3D30	4.16	29.8	4.48	32.0	4.57	57.6
3D30+2	3.17	26.1	3.68	28.2	4.16	59.1

**Table 10:** Statistical analysis results.

Type of Strength	Direction	Shapiro–Wilk Test			Levene’s Test	One-Way ANOVA	Kruskal–Wallis Test
		3DREF	3D30	3D30+2			
Compressive strength	Perpendicular	0.0283	0.4447	0.6947	0.3716	0.0011	0.0338
	Longitudinal	0.3452	0.2284	0.5262	0.2020	7.18 × 10^−8^	0.0017
Bending strength	Perpendicular	0.2342	0.3125	0.4723	0.7177	0.0228	0.0510
	Longitudinal	0.3244	0.4068	0.9676	0.4471	0.2230	0.2881

## Data Availability

The original contributions presented in this study are included in the article. Further inquiries can be directed to the corresponding author.
